# Major Biological Control Strategies for Plant Pathogens

**DOI:** 10.3390/pathogens11020273

**Published:** 2022-02-19

**Authors:** Manisha Arora Pandit, Jitendra Kumar, Saloni Gulati, Neeru Bhandari, Poonam Mehta, Roma Katyal, Charu Dogra Rawat, Vachaspati Mishra, Jasleen Kaur

**Affiliations:** 1 Department of Zoology, Kalindi College, University of Delhi, Delhi 110008, India; manishaarorapandit@kalindi.du.ac.in; 2 Bangalore Bioinnovation Centre, Life Sciences Park, Electronics City Phase 1, Bengaluru 560100, India; director@bioinnovationcentre.com; 3 Department of Botany, Dyal Singh College, University of Delhi, Delhi 110003, India; salonigulati@dsc.du.ac.in (S.G.); neerubhandari@dsc.du.ac.in (N.B.); poonammehta@dsc.du.ac.in (P.M.); romakatyal@dsc.du.ac.in (R.K.); 4 Department of Zoology, Ramjas College, University of Delhi, Delhi 110007, India; cdrawat@ramjas.du.ac.in

**Keywords:** plant pathogen, biocontrol, microbes, AMF, bacteriophages, microbiome, sustainable strategies

## Abstract

Food security has become a major concern worldwide in recent years due to ever increasing population. Providing food for the growing billions without disturbing environmental balance is incessantly required in the current scenario. In view of this, sustainable modes of agricultural practices offer better promise and hence are gaining prominence recently. Moreover, these methods have taken precedence currently over chemical-based methods of pest restriction and pathogen control. Adoption of Biological Control is one such crucial technique that is currently in the forefront. Over a period of time, various biocontrol strategies have been experimented with and some have exhibited great success and promise. This review highlights the different methods of plant-pathogen control, types of plant pathogens, their modus operandi and various biocontrol approaches employing a range of microorganisms and their byproducts. The study lays emphasis on the use of upcoming methodologies like microbiome management and engineering, phage cocktails, genetically modified biocontrol agents and microbial volatilome as available strategies to sustainable agricultural practices. More importantly, a critical analysis of the various methods enumerated in the paper indicates the need to amalgamate these techniques in order to improve the degree of biocontrol offered by them.

## 1. Introduction

A large amount of crop loss occurs each year during both pre and post-harvest stages due to pathogen infestation that involves a wide variety of pathogens ranging from viroids and viruses to prokaryotic bacteria, eukaryotic fungi, oomycetes, and nematodes. These plant pathogens are highly persistent in their attack and induce direct and indirect losses to the tune of 40 billion dollars worldwide [1]. Over the last decade some very important aspects of microbial applications in crop disease mitigation have been discussed [2,3,4] as methods of sustainable agriculture. However, their field application is still inadequately worked out.

Given the paramount importance of the methods for controlling plant pathogens and diseases caused by them to improve productivity not only in terms of food but also for other materials obtained from plants like fibre, timber, oils, medicines, etc. and to meet the food demands of the exponentially growing world population, food production needs to increase by 70% by the year 2050 to address the internationally growing food security concerns [5]. It is high time when we need to shift to sustainable methods of agriculture so as to reduce biodiversity loss and greenhouse gas emissions that are currently placed at 60% and 25%, respectively [1]. At present, most of the methods employed for plant protection from pathogens primarily involve the use of antibiotics and chemicals [6]. Even though these shotgun [6] methods deliver immediate protection, they ultimately lead to resistance and bioaccumulation of harmful chemicals in the crop systems. It is these drawbacks that emphasize the importance of sustainable and environment-friendly crop management practices to control diseases [7]. Such practices help to improve the quality and quantity of agricultural produce that also includes organic crops. The organic systems exploit various naturally occurring plant protection resources like micro- and macro-flora and fauna found in the soils, protective products made from plant extracts, use of physical methods like weeding, mulching, and choice of cultivars, etc. to support organic produce [8]. Hence ingredients of organic agricultural practices can serve as model tools in establishing sustainable methods of agriculture as a whole. Overall, due to the growing concerns of environmental pollution and ecological toxicity resulting from the indiscriminate use of chemical formulations, there is an immediate need to base modern plant protection strategies on natural resources [1].

The terms biological control or biocontrol used extensively in scientific literature, cause tremendous confusion. Biological control, in its most basic form, is the employment of any living organism to combat a specific plant disease or pest through parasitism, antibiosis, or competition for resources or space [9]. In order for a disease or pest to thrive on a plant, three important criteria need to be fulfilled. These include the invader (the plant pathogen or pest), the environment, and the plant itself. Therefore, there are complicated processes at several levels that not only produce the diseases and pests, but also modulate them [10]. As a result, a broader definition of biological control is necessary, one that encompasses all levels, to realize its full potential in disease and pest management. This broad definition will involve the use of species and their byproducts to manage pests and diseases in crops, either via hostile reactions or through the development of immunity against them [11]. Despite extensive studies devoted to field trial effectiveness of biocontrol agents (BCAs), this area is restricted due to changes in ecological characteristics, such as the host‘s physiological and genetic state, climatological circumstances, and other factors that enhance the variability of the desired BCA impact [12,13,14]. As a result, most biocontrol applications are limited to greenhouse crops, where environmental conditions are monitored and supervised [15]. It is suggested here that combinations of BCAs and fungicides are able to control pathogens more effectively [16]. However, this area is entirely barren and extensive research studies need to be conducted to come out with meaningful conclusions. In the current study, we examine several biocontrol approaches against plant diseases and upcoming strategies that offer improved biocontrol potential against a diverse population of pathogens that might possibly assist in the attainment of long-term sustainability goals.

## 2. Plant Disease Management

### 2.1. Chemical Control

There has been high dependence on chemicals to control diseases and pests in agriculture and even today, they continue to remain the main component of Integrated Pest Management (IPM) as demonstrated by the ever-increasing use of fungicides since the 1960s [17]. These chemical formulations, even though crucial to prevent large scale losses and spread of diseases in crops, come with several drawbacks such as, ecotoxicity, bioaccumulation, adverse effects on nontarget plants and animals, and human health. Exposure to these chemical-based pesticides, fungicides, etc. is known to cause various types of cancers, respiratory disorders, and hormonal imbalances in humans [16]. Apart from these, data from FAO-WHO and US Food and Drug Administration shows that persistent organic pollutants (POPs) do not degrade easily and remain deposited on fruits and vegetables, ultimately entering animal-based food sources like dairy products, poultry, and meat [6]. Furthermore, the use of chemical pesticides has led to continued rise of resistant pathogens resulting into reduced efficacy of most chemical control methods [16].

### 2.2. Resistant Varieties

The process of crop selection and plant breeding are well known and proven criteria that are applied in agriculture to improve crop varieties and produce disease-resistant cultivars. These practices are used even today and have proven to be beneficial in the fight against various types of disease-causing plant pathogens [6]. The genetic route is one of the most favored biotechnological applications in our never-ending strive to increase food production. Genetically modified (GM) varieties are not only disease resistant, but also produce better quality crops and greatly reduce the need for external inputs of costly chemicals, thereby making their production economically viable. Despite these advantages, GM crops require approval from regulatory agencies at a high cost and are not readily accepted by the consumers. Moreover, these crops can also exhibit susceptibility to pathogens within a few years of their cultivation due to a number of causes like mutations occurring in the targeted pathogens, reduction in field resistance due to various recombination events, and lack of genetic uniformity within the GM crops [18]. Many crops have shown indications of resistance breakdown, including rice blast resistance, cotton leaf curl disease, grapevine downy mildew, and yellow wheat rust [18]. Nevertheless, encouraging results are being achieved in the labs by using genome editing by CRISPER/Cas9 and insertion of gene cassettes using intragenic technologies and it is expected that in the near future, these approaches may be the way forward and can be used at par with conventional plant breeding technologies [6]. Other breeding methods involving gene pyramiding, gene rotation, and multiline varieties also offer advantages in controlling resistance. It is imperative that newer and better biotechnological tools are developed and applied in order to accelerate the production of improved disease-resistant cultivars so as to manage the newer aggressive pathogens [18].

### 2.3. Biological Control

Among the non-chemical methods of pest and pathogen control, biological control or biocontrol seems to be the most suited for organic cultivation. It is environmentally safe, sustainable, economically viable, and highly specific (Table 1). A number of such methods are currently being employed, like the use of naturally occurring soil microbes against various pests and pathogens [2]. A deeper understanding of the relationships between plants and pathogens along with the environmental factors prevalent in a particular area needs to be understood prior to biocontrol implementation, particularly under widespread disease conditions. In plant pathology, biocontrol is defined as the interaction of numerous environmental elements with the goal of reducing the negative impacts of harmful species while promoting the growth of beneficial crops, helpful insects, and microbes [19]. Biological control is dependent on numerous agonistic and antagonistic interconnections between plants and microbes living in the rhizosphere and phyllosphere [20] and their application to minimize disease and subdue pests. Organisms from the rhizosphere can be harnessed from the surrounding environment (the black box approach) or can be introduced into the field from external sources (the silver bullet approach). It is beneficial to apply a consortium of microbes with collaborative properties rather than relying on a single organism since microbial consortia make up a stable rhizosphere that offers more effective control against pathogens [21]. Apart from microbial applications, the utilization of other plant products like extracts, biofertilizers, and biopesticides, natural enemies of pests and pathogens, and gene products also aid in carrying out biological control [6].

## 3. Types of Plant Pathogens

Plant pathogens are divided into three categories namely necrotrophs, hemibiotrophs, and biotrophs depending on the way they obtain energy from the plants [6]. These interconnections in turn influence the way the plant responds to the pathogens [6,54].

### 3.1. Biotrophic Pathogens

Biotrophic plant pathogens obtain their nourishment from living cells of the host plant with the help of complex mechanisms to access plant resources. They share a close relationship with the plants’ living tissue to the extent that some of the biotrophs have lost the ability to grow on non-living artificial media and have coevolved as obligate biotrophs. Examples include *Uromyces fabae* that causes rusts and *Blumeria (Erysiphe) graminis* that causes powdery mildews [55,56]. The non-obligate biotrophs on the other hand can be grown on artificial media, are not saprophytic, and restrict injury only to the host cells. Biotrophs form hyphae/ haustoria that penetrate the host cell wall but not its plasma membrane. The plasma membrane at these points invaginates and gives rise to a perihaustorial/ peri arbuscular membrane where nutrient exchange takes place [57]. Effector molecules are released by the pathogen that further helps in the invasion of the host genotype [56,58,59,60]. Other examples include *Ustilago maydis*, which causes corn smut and *Cladosporium fulvum* that causes tomato leaf mold, do not form haustoria and nutrient exchange between the plant and the microbes is carried out via apoplast [61].

### 3.2. Necrotrophic Pathogens

Unlike biotrophs, the necrotrophic microbes are opportunistic, unspecialized pathogens that kill the host rapidly and sustain on its remains [62,63]. They do not form haustoria and enter the plant via naturally found openings or wounds and secrete lytic enzymes and phytotoxins. They can be easily grown on artificial media. Necrotrophic pathogens include bacteria, fungi, and oomycetes that mainly attack young, weak, and damaged plants and are capable of a saprotrophic mode of existence [63,64]. Both bacterial and fungal necrotrophs follow similar patterns of infection that involve attachment, host penetration, and subsequent necrosis and decay of plant tissues. Some examples of fungal necrotrophs are *Cochliobolus* that causes corn leaf blight, *Alternaria* that causes early blight of potato and *Botrytis* that causes grey mold [6,56,65,66]. Mechanisms of plant immunity against these pathogens are in the form of phytohormones, pathogenesis proteins and secondary metabolites [63]. Some of the important cash crops that are infected by necrotrophic fungi like *Fusarium* and *Rhizoctonia* include wheat, maize, and rice [67,68,69]. Even if a percentage of the crop genotype does not respond to the toxins produced by the necrotrophic fungi and evades necrosis, these pathogens are still capable of inflicting a much greater loss of productivity and overall destruction in comparison to the biotrophs [63].

### 3.3. Hemibiotrophic Pathogens

Hemibiotrophic pathogens are an interesting group of pathogens as they display characters of both biotrophs and necrotrophs and are capable of switching between the two modes. The transition from the asymptomatic biotrophic phase to the destructive necrotrophic phase is accompanied by suppression of the host’s immune response at the required time resulting in extensive damage to the host leading to its decay and death [6]. Hemibiotrophic characteristics are shown by fungi like *Magnaporthe grisea*, *Phytophthora*, *Pythium*, *Fusarium*, *Colletotrichum* and *Venturia*, and the bacterium *Pseudomonas syringae* all of which are capable of a prior biotrophic existence with the host but ultimately shift to a necrotrophic mode of nourishment by killing the host cells [56,58,59,60,64,70,71].

## 4. Biocontrol Management

### 4.1. Microbial Biocontrol

The rhizosphere is the soil area that surrounds the roots and is composed of microbes capable of repressing plant pathogens. It, therefore, aids in providing natural protection to the plants against a variety of organisms either directly by synthesizing metabolites antagonistic towards the pathogens or indirectly by suppressing pathogen growth and improving the host‘s defense mechanisms. Antibiosis caused by the release of antibiotics, organic compounds, toxins, and various hydrolytic enzymes like beta-xylosidase, chitinase, pectin methylesterase, β-1,3-glucanase, etc. is one of the mechanisms employed by the rhizosphere microbial population to carry out the destruction of the pathogen including disintegration of the glycosidic linkages in its cell wall [6]. Plant growth-promoting rhizobacteria (PGPR) residing in the rhizosphere also perform biocontrol by reducing the incidence of plant disease thereby assisting in plant growth. The PGPR also promote antibiosis, competition, production of metabolites that induce systemic acquired resistance (SAR) and induction of systemic resistance (ISR), parasitism, production of hydrolytic enzymes such as cellulase, glucanase, chitinase, and protease that break down the cell wall along with a number of antibiotics like oomycin A, 2,4-diacetyl phloroglucinol (DAPG), pyoluteorin etc against the pathogens [72]. For example genus, *Serratia* belonging to Enterobacteriaceae is a PGPR that produces secondary metabolites having attractive biocontrol properties [73].

Rhizobia are symbiotic microbes found on the roots of leguminous plants that not only play an important role in nitrogen fixation but also in biocontrol. They promote plant growth by secreting antibiotics, mycolytic enzymes, siderophores, and hydrocyanic acid (HCN) that prevent the growth of pathogenic fungi belonging to genera like *Fusarium*, *Rhizoctonia*, *Sclerotium*, and *Macrophomina*. They enhance plant immunity by increasing the expression of defense-related genes and instigating systemic resistance.

Seed quality can be improved by bacterization with the correct rhizobial strain to cause activation of various enzymes involved in isoflavonoid and phenylpropanoid pathways, accumulation of phenolic compounds and isoflavonoid phytoalexins that enhance the biocontrol capability of the cultivars thereby improving plant growth and productivity [74]. Examples of protection by rhizobia can be seen in the use of a colloquium of *Pseudomonas* strains that were isolated from potato phyllosphere and rhizosphere and used to fight the late blight of potato caused by *Phytophthora infestans*. The colloquium of different strains proved to be far more effective compared to the use of individual strains [75]. Plant disease management also engages endophytes as biocontrol agents. These microbes can reside asymptomatically in different parts of a plant like a shoot, leaves, or roots [76,77,78,79,80,81]. Potential antagonistic strains of endophytes can be screened for biocontrol capability as all strains do not exhibit similar activity. This was exhibited by Gonthier et al. [82] on the use of *Suillus luteus* against the fungal pathogens *Heterobasidion irregular* and *Heterobasidion annosum* that infect Scots pine (*Pinus sylvestris*) that resulted in diminished susceptibility to only *H. annosum*, and not to *H. irregular*. They can also be engaged as control methods against threats such as the spotted lanternfly that causes severe economic loss in North America [83,84]. Endophytes use varied mechanisms like lytic enzymes, activation of host defenses, synthesis of antibiotics, and mycoparasitism against pathogens. In-depth research on their biocontrol activity is much required in order to exploit their full potential as future disease and pest management agents [85,86].

### 4.2. Fungal Biocontrol

Apart from their ability to improve nutrient uptake and nitrogen use in plants, fungi also have biocontrol capabilities. They can aid in the fight against pests like nematodes and microbial pathogens that infect various parts of the plant such as roots, foliage, and fruits. They offer protection against diseases with the help of processes like mycoparasitism, competition for resources with pathogens, antibiosis, conferring ISR to the host plant, and mycovirus mediated cross-protection or MMCP [87]. Some of the well-known fungal biocontrol agents include the *Trichoderma* species, ectomycorrhizas, arbuscular mycorrhizas (AMF), yeasts, and endophytes. Even the nonvirulent strains of certain pathogens can utilize hypovirulence-associated mycoviruses in order to function as biocontrol fungi [88]. With improved biotechnological and genetic advances, it is not only possible to introduce beneficial fungal genes into the genomes of the host plants but also to interrupt or overexpress these genes in order to improve biocontrol ability [88]. A review by Thambugala et al. [89] provides a comprehensive list of fungal biological control agents that were used against fungal plant pathogens according to modern taxonomic concepts, and clarifies their phylogenetic relationships. Furthermore, they clarify that this is important in view of the wrong names are frequently used in the literature of biocontrol. They list details of some 300 fungal antagonists belonging to 13 classes and 113 genera together with the target pathogens and corresponding plant diseases. According to them, *Trichoderma* is identified as the genus with greatest potential of biocontrol and it comprises 25 species as biocontrol agents that have been used against a number of plant fungal diseases. In addition, nine more genera were recognized by them as significant in this regard that comprise five or more known antagonistic species, namely, *Alternaria*, *Aspergillus*, *Candida*, *Fusarium*, *Penicillium*, *Pichia*, *Pythium*, *Talaromyces*, and *Verticillium*. Majority of the plant growth-promoting fungi (PGPF), viz., *Trichoderma*, *Penicillium*, *Aspergillus* and *Fusarium* spp. are reported for their abilities to stimulate the plant immune responses upon enemy attack and are considered as one of the safest modes for induced systemic resistance (ISR) and growth promotion in crop plants [90,91]. In addition, PGPFs are also known for being beneficial to plants in reducing the impacts of various fungi, bacteria, viruses and nematodes [91] by eliciting ISR. Trichoderma species are soil-borne filamentous fungi known for its utility in many plant health benefit applications [92]. Its strains deploy a complex mechanism in pathogen control that includes colonizing the soil and root of the host, inhabiting a physical space and evading the multiplication of the phytopathogens while concomitantly producing cell wall-degrading enzymes, antimicrobial metabolites to kill the pathogens, inducing plant defense mechanisms, promoting plant development and improving plant tolerance to biotic and abiotic stressors [93].

### 4.3. Plant Virus and Biocontrol

Qu et al. [94] have elucidated the effects of a single-stranded DNA virus, Sclerotinia sclerotiorum hypovirulence-associated DNA virus 1 (SsHADV-1) that infects the fungus *Sclerotinia sclerotiarum*, a disease causative agent of many crops [94]. Qu et al. [94] have further elucidated the altered expression of phenotype related genes upon SsHADV-1 infection by using digital RNA sequencing.

Some common and well characterized useful viruses that harbour the plants are those that are known to enhance the beauty of ornamental plants. Tulip breaking virus was the first of this lot. However, many other prized ornamentals owe their value to some extent to the viruses that infect them [95]. Other examples of beneficial plant viruses include several acute viruses, such as Brome mosaic virus, family Bromoviridae, Cucumber mosaic virus, family Bromoviridae, Tobacco rattle virus, family Virgaviridae, and Tobacco mosaic virus, family Virgaviridae, which confer tolerance to drought and freezing temperatures in various crops, and persistent viruses, such as White clover cryptic virus (family Partitiviridae), which can suppress nodulation in legumes during proper supply of nitrogen [96]. Roossinck, [96] elaborates further by stating that the mild symptom causing plant virus strains have been used for cross-protection against more severe strains and this attribute is utilized in pathogen-derived transgenic resistance strategies.

A good number of literature reveal that plant infected with viruses do not show any apparent ill effects on their hosts in the beginning [96,97]. However, their persistence as displayed by the virus families Chrysoviridae, Endornaviridae, Partitiviridae and Totiviridae that are a group of the most common viruses found in wild plants, has got significance from scientific perspectives pertaining to biocontrol of plant diseases [97]. These viruses are considered as having very long relationships with their plant hosts. Persistent viruses are also common in crops, including peppers, rice, beans, carrots, figs, radish, white clover, melons, barley and avocados [98].

Plant viruses are controlled either by host resistance, e.g., Plum Pox Virus (PPV) by activation of members of a cluster of meprin and TRAF-C homology domain (MATHd)-containing genes that were designated as possible PPV resistance genes [99], mild strain cross protection, e.g., Pepino Mosaic Virus (PepMV)-based cross protection in the crops [100], or by biocontrols of their insect vectors, e.g., parasitoids of mealybugs that vector GLRaV-3. The latter approach has been tested for vine mealybug, *Planococcus ficus* that feeds through a membrane feeding system on GLRaV-3, which was blocked with some blocking molecules in a test study for such molecules tapping the feeding membrane system of the parasitoid vector [101]. Pechinger et al. [100] have provided a detailed list of protective virus isolates and their respective challenging isolates tested for their mild strain cross-protection capabilities. In case of host resistance, host R genes typically induce race-specific resistance in response to the Avr genes of pathogens [102,103]. During plant–virus interactions occurring in a single cell, an R gene triggered HR response is vital that kills infected cells and restricts the viral invasion and this phenomenon is associated with several molecular events, such as the activation and expression of salicylic (SA), jasmonic acid (JA), mitogen-activated protein kinase signaling [103], calcium ion influx, callose deposition at the plasmodesmata, membrane permeability modification, pathogenesis-related (PR) protein expression, and immediate accumulation of reactive oxygen species and nitric oxide [104].

### 4.4. Arbuscular Mycorrhizal Fungi (AMF) Biocontrol

A number of studies lay emphasis on the biocontrol abilities of Arbuscular mycorrhizal fungi (AMF) as they have been shown to reduce the incidence of fungal diseases and nematode attacks on host plants by 30 to 42% and 44–57%, respectively [2,3,105,106]. The biocontrol properties of AMF are broad-spectrum and more pronounced against fungal root pathogens in comparison to the shoot ones [107,108]. AMF offers defense against a number of fungal pathogens belonging to the genera *Colletotrichum*, *Alternaria*, *Erysiphe*, *Gaeumannomyces*, *Macrophomina*, *Botrytis*, *Rhizoctonia*, *Fusarium*, *Cylindrocladium*, *Sclerotium*, and *Verticillium*. On the other hand, they do not offer much protection against a large number of bacterial and viral pathogens but some bacteria like *Pseudomonas syringae pv. glycinia* that causes bacterial blight on soybean can be checked by AMF. In the case of viral pathogens, the presence of mycorrhizal fungi seems to increase the damage caused by viral infections [87] as seen with Tomato spotted wilt virus (TSWV) [109], Potato virus Y [110], Citrus tristeza virus and Citrus leaf rugose virus [111] and Tobacco mosaic virus [112]. Therefore the role of AMF against viral pathogens is largely unclear and mostly points towards a supportive influence resulting in intensified disease rigor [109,113]. Moreover, reduced colonization and spore formation is shown by the AMF when the host plant is infected with a viral pathogen like the yellow mosaic virus [114].

### 4.5. Biocontrol Yeast

Yeasts such as *Aureobasidium pullulans*, *Cryptococcus albidus*, *Candida oleophila*, *Saccharomyces cerevisiae*, and *Metschnikowia fructicola* are currently being employed as biocontrol agents as they are effective adversaries of various plant pathogens. Yeasts are a category of unicellular fungi that grow in most environments, have simple culture needs and few if any biosafety concerns. They apply competition, volatiles, enzymes and toxins, mycoparasitism, and initiation of immune response mechanisms for plant protection. Due to these properties, they can be exploited as biocontrol effectors but a paucity of studies on their role limits their full utilization [115]. Yeasts are known to exert their biocontrol activity through Phage based competition, enzyme secretion, toxin production, volatiles, mycoparasitism, induction of resistance activity [115]. Ferraz et al. [116] have extensively listed the success cases of using yeasts to antagonize the spoilage of fruits by filamentous fungi. Thambugala et al. [89] have prepared an exhaustive list of some commercialized fungal biocontrol agents for plant fungal diseases and their specifications, that is suggested for further reading. Some important yeast species, such as *Candida oleophila*, *Aureobasidium pullulans*, *Metschnikowia fructicola*, and many others are reported to have been registered as biocontrol agents and have been suggested to having potential for being utilized as commercial biocontrol agents [115]. Di Canito et al. [117] suggests that Saccharomyces and non-Saccharomyces yeasts as potential antagonists against phytopathogenic fungi of the genera *Penicillium* and *Aspergillus* and the species *Botrytis cinerea* on table grapes, wine grapes, and raisins. They suggest that several non-conventional species are largely unexplored till date in both basic research and for their possible utilization in commercialization. They further say that this group constitutes a huge, untapped reservoir of yeasts having potential for biotechnological innovations constituting selection of species and strains with new metabolic traits, such as the secretion of proteins, adhesiveness, antimicrobial properties, etc. that are required for yeasts to manifest their applications as biocontrol agents. Application of yeasts in prevention of infections represent a new strategic frontier for maintaining the post-harvest quality of table and wine grapes [117]. The genomes of several non-conventional yeast species have been completely sequenced and their number is growing continuously Wendland [118]. Thus, expected novel methods for the genetic analysis and their further modifications in yeasts, as well as their genomic and post-genomic analysis before and after such modifications, will represent a platform for understanding the molecular mechanisms underlying both the simple and complex biological features that are supposed to be useful for the development of new and eco-compatible applications.

### 4.6. Phage-Based Biocontrol

Phages have been in use as biocontrol agents against bacterial pathogens for a long time. The earliest study demonstrating their biocontrol ability was done by Mallmann and Hemstreet in 1924 [119] in which they isolated *Xanthomonas campestris* pv. *campestris* from plant tissues suffering from the cabbage-rot disease. Future studies showed that phages could inhibit soft-rot caused by *Pectobacterium carotovorum* subsp. *carotovorum* in carrots [120], a bacterial spot of tomato by *X. campestris* pv. *vesicatoria* [121] and *Pectobacterium atrosepticum* in potato slices [122]. More recent explorations into phage biocontrol usage have focussed on improving their durability under field conditions [123]. Exploring the use of phage cocktails and systemic acquired resistance activator in disease management against *X. citri subsp. citri* and *Xanthomonas axonopodis* pv. *citrumelo* that causes citrus bacterial canker and citrus bacterial spot respectively showed positive results in field trials [124]. On the other hand, some studies showed a better disease management response in laboratory-based bioassays rather than in field trials, like in the case of phage treatment against *Pseudomonas syringae pv. porri* that causes bacterial blight of leek [125]. However various economically significant bacterial pathogens like *Xanthomonas* spp. and *Pseudomonas syringae* can be effectively controlled by phages. Peptidoglycan hydrolases, lysins from phages Atu_ph02 and Atu_ph03 are capable of blocking cell division in *Agrobacterium tumefaciens* (causes crown gall disease) resulting in its lysis [126]. Other lysins from CMP1 and CN77 phages have also shown lytic capacity against *Clavibacter michiganensis* subsp. michiganensis, that causes bacterial wilt and canker of tomato [127]. The incorporation of phage lysins into transgenic crops can aid in their easy application and overcome production issues [127]. Mostly, the application of phages and phage lysins in plant disease management is a progressive step and has shown positive outcomes in a number of instances. Focus now needs to be on developing better delivery methods and guaranteeing a longer shelf life for the phage and its enzymes on the host plant [7].

### 4.7. Natural Compounds against Plant Diseases

Bioactive natural compounds can be of plant or animal origin and are capable of controlling plant diseases thereby promoting plant growth. A number of bioactive molecules belonging to phenolic, terpenoid, or alkaloid categories [128] such as chitin, laminarin, allicin, terpenes, chitosan, naringin, and carrageenans have been identified for use as biopesticides in organic cultivation. Allicin, acquired from garlic exhibits antibacterial and antifungal properties under field conditions [129,130,131], garlic juice inhibits the growth of a number of bacteria belonging to the genus *Pseudomonas*, *Agrobacterium*, *Xanthomonas*, and *Erwinia* and fungi *Cercospora arachidicola*, *Botrytis cinerea*, *Rhizoctonia solani*, *Alternaria alternata*, *Fusarium moniliforme*, *Colletotrichum coccodes* [132,133]. Naringin (40,5,7-trihydroxyflavanone-7-β-d-α-l-rhamnosyl(1-2)-β-d-glucoside) is another potent bioactive molecule found in seeds and pulp of grapefruit [134] that displays effectiveness against fusariosis, alternariosis, and gray mold infections in soybean, ornamental plants, and vegetables such as potato [135,136,137]. Tea tree oil (*Melaleuca alternifolia* L.) contains terpenes like terpinen-4-ol, gamma-terpinene, 1,8-cineole and exhibits strong antimicrobial properties against a variety of bacteria and fungi. It is particularly effective against *Bremia lactucae* and downy mildew that attack lettuce [138,139,140]. At times the use of bioactive compounds like garlic pulp is more beneficial than synthetic compounds like azoxystrobin as seen in the case of sweet pepper plants [133]. Chitin which is the second most abundant polysaccharide in nature and a component of the fungal cell wall and exoskeleton of crustaceans and insects shows bioactivity against a number of bacterial, viral, and fungal pathogens [141]. It is known to have a strong antifungal influence against soil-borne pathogenic fungi that infect soybean [135] and is a fungal microbe-associated molecular pattern (MAMP) molecule that is able to activate immune responses in the host plant [1]. It can be isolated using enzymatic reactions and chitosan distillation [142]. Bioactive compounds, therefore, show a variety of modes of action not only to limit pathogen growth and multiplication but also inactivation of the host defense response [143]. They usually act via binding to the membrane receptors on plants and produce a signal that is capable of initiating an immune response.

### 4.8. Algal and Cyanobacterial Biocontrol

Apart from being an abundant source of vitamins, saccharides, enzymes, amino acids, phytohormones and elements like molybdenum, boron, manganese, iron, iodine, and zinc, algae and cyanobacteria extracts are a rich source of bioactive elicitors [144,145] with antifungal, antiviral and antibacterial properties [146]. These extracts are usually applied in agriculture to improve productivity and plant vitality. Use of extracts from the algae *Sargassum filipendula*, *Ulva lactuca*, *Caulerpa sertularioides*, *Padina gymnospora* and *Sargassum liebmannii* ease symptoms of fungal infection on tomato produced by *Alternaria solani* and *Xanthomonas campestris* pv. *vesicatoria* [147,148]. Studies on tomato seedlings infected by *Macrophomina phaseolina* showed improvement after the application of *Kappaphycus alvarezii*. The algal action was propagated through improved levels of phytohormones (salicylic acid, indole-3-acetic acid and abscisic acid), transcription of PR-1b1, PR-3, and PR-4 genes, and the cytokinin zeatin [149]. The activity of polyphenol oxidase and peroxidase enzymes important in plant defense in tomatoes was also shown to improve when extracts from *Cystoseira myriophylloides*, *Laminaria digitata* and *Fucus spiralis* were utilized against *Verticillium dahliae* wilt [150].

Cyanobacteria have been applied against plant pathogens both at the levels of soil and leaves. Employing *Nostoc entophytum* and *Nostoc muscorum* in the soil against *Rhizoctonia solani* greatly enhanced seedling endurance along with improving root and shoot dry weight and plant length [151]. In tomato, application of *Nostoc linckia* in soil against *Fusarium oxysporum* f. sp. *Lycopersici* decreased wilt while an improved state of similarly infected seedlings of tomato was observed with *Nostoc commune* [152,153]. Usage of cyanobacteria, *Anabaena* sp. on zucchini cotyledons infected with powdery mildew (*Podosphaera xanthii*) resulted in enhanced enzymatic activity of peroxidases, endochitinase, chitin 1,4-β-chitotriosidase, β-N-acetylhexosaminidase, and β-1,3-glucanase [154]. Similar enzymatic activation was observed by Prasanna et al. [155] upon employing a biofilm composed by *Anabaena* sp. on maize roots and shoots. Cyanobacteria, like algae are also capable of high polysaccharide production in response to various categories of plant pathogens but there is a lack of data which limits their use as biocontrol agents [156,157,158].

## 5. Emerging Biocontrol Strategies

### 5.1. Microbial Volatilome and Its Role in the Biological Control

One of the most resilient and encouraging solutions in biocontrol approaches is the employment of microorganisms as biological control agents (BCAs). Among the several microbiological strategies used by BCAs, the production of volatile organic compounds (VOCs) is a method that is helpful in situations where the straightforward association between the pathogen and its competitor is not possible. All living forms synthesize VOCs and these can be exploited for usage in biocontrol of plant pathogens like bacteria, oomycetes, and fungi. VOCs are a sustainable preference for synthetic fungicides due to their ease of application, low residue deposition in the environment and on crops, and their biocontrol efficacy [159]. According to Tahir and colleagues [160], VOCs produced by *Bacillus* species are known to function at a number of levels against the tobacco wilt agent *Ralstonia solanacearum*. In vitro studies indicate that *Bacillus* volatile compounds reduced Ralstonia growth and viability and caused significant problems in cell integrity and motility in addition to considerable alterations in *Ralstonia* genes expression that controls disease progression [160]. Furthermore, tobacco plants treated with *Bacillus* emissions and purified detected VOCs elevated transcription levels in critical defense-related genes such NPR1 and EDS1, leading to inhibition of systemic resistance [160]. It’s possible that bacterial volatiles has a role in *Bacillus* reported biocontrol properties both directly and indirectly, and that bacterial VOC bouquets function as multifactorial, sequential, or simultaneous signals on pathogens and hosts [161].

### 5.2. Microbiome-Based Solutions for Plant Protection

New findings demonstrate a remarkable microbial diversity among all plants, as well as unique phytopathogen antagonistic bacteria. Mosses, which are the world’s oldest land plants, exhibit a unique microbial diversity, and their ecology allows them to contain a large number of enemies [162,163]. Apart from mosses, medicinal and endemic plants are also likely sources of rare biodiversity and enemies. A characteristic acquired by them due to their unique metabolism, that alters the architecture of the plant microbiome [164,165]. We expect endophytes, particularly seed endophytes, to serve as sources for novel biocontrol agents as a result of new discoveries. Until now, bacteria and fungi have been used mostly for biocontrol. Archaea have just recently been recognized as part of the plant microbiome [166]; their effects on plants and potential for biocontrol are unknown. Microbial invasion can affect the network of microorganisms that are linked with plants. These network models of soil and plant microbiomes can be interpreted for biocontrol and present new prospects for disease management. While single organisms were commonly utilized in the past, their effects were often uneven, microbiome-based biocontrol techniques are now possible [167].

In the future, microbial consortia and biocontrol agents can be employed to improve biodiversity associated with crops via microbiome engineering so as to achieve definitive microbiome outcomes as desired [168]. Crop-specific biological consortia can be assembled from a pool of selected biocontrol agents in this setting. Taking a holistic approach and incorporating microbiome-based solutions allows for targeted and predictive biocontrol measures. Furthermore, integrated breeding and biocontrol measures are essential to sustain ecosystem variety and health. These systemic techniques are necessary to prevent further biodiversity losses and promote sustainable agriculture operations [167].

### 5.3. Phage Cocktail

Phage cocktails are a feasible option for controlling a variety of plant diseases; however, further study and solutions to technical obstacles are needed to achieve successful biocontrol. Thorough knowledge of interactions between plant, phage, and pathogen is required since the habitat of each plant system is unique and complex. This can only be achieved by conducting more extensive field experiments, as in vitro and in vivo tests under laboratory conditions do not accurately reflect the real circumstances in the field. More advanced protective formulations are needed to ensure the survival of phage mixtures during long-term storage under ambient conditions. The use of already existing phages from the phyllosphere can provide better protection against the phytopathogens in that environment. To improve phage persistence in the phyllosphere, light-absorbing compounds and/or protective formulations could be added to phages that have evolved to resist UV-induced damage. Synthetic phage cocktails with customized host ranges can also be created using genetically engineered phages. More research is needed in order to obtain well-characterized phages with defined and configurable host ranges. Finally, due to the great diversity of phytobacteria, a single universal phage cocktail for all diseases is not viable. Designing tests that can identify the disease-causing bacteria and its antagonistic phage can greatly aid in its control. To date, no such simple and economical option is either available or implemented [169].

### 5.4. Genetically Modified Biocontrol Agents

To improve the efficacy of BCAs, techniques for genetic engineering of all organisms can be used. *Rhizoctonia solani* infection in beans can be brought under control by transferring a gene coding for the enzyme chitinase from *Serratia* to a *Pseudomonas* endophyte [170]. While transferring a gene for glucanase to *Trichoderma* produced resistance to pathogens such as *Rhizoctonia, Rhizopus*, and *Pythium* [171]. Cloning of 2,4-diacetylphloroglucinol (2,4-DAPG) biosynthetic locus phlACBDE from strain CPF-10 into a mini-Tn5 transposon by Zhou et al. [172] into a mini-Tn5 transposon and its insertion into the chromosome of *Pseudomonas fluorescens* P32 improved resistance of wheat to *Gaeumannomyces graminis var. tritici* and tomato to *Ralstonia solanacearum* bacterial wilt. Regardless of the findings of this research, these newly produced BCAs are subject to the same restrictions that apply to organisms that have been genetically changed using recombinant DNA technology. Clermont et al. [173] employed genome shuffling to create superior *Streptomyces melanosporofaciens* EF76 biocontrol strains. Four strains with improved antagonistic activity against the potato diseases *Streptomyces scabies* and *Phytophthora infestans* were isolated after two rounds of genome shuffling. Biological control ability can also be improved by employing chemical mutagenesis. Examples include the use of nitrosoguanidine mutagenesis in *Pseudomonas aurantiaca* B-162 to produce a strain with better phenazine synthesis leading to improved biocontrol activity [174] and *Trichoderma harzianum* strains that exhibited enhanced biocontrol ability after UV mutagenesis [175]. The addition of the required mutation can at times produce altered gene expression in non-targeted genes resulting in undesired effects. These constraints can be overcome using more recently established genome editing approaches. We can insert mutations into specific regions in the genome with high precision and efficiency using techniques like Crispr/Cas [176]. Another benefit is that mutations can be induced in numerous genes at the same time, which will aid in determining the role of different genes in biocontrol [177]. The gene editing approach could also help in commercialization of BCA’s through ease of regulatory clearances.

### 5.5. Microbiome Engineering

Many researchers suggest the microbiome to be representative of a “second genome” [178,179], but some prefer the term “holobiont” to describe the variety of microbes linked with plant and animal hosts [180]. Microbiome engineering has the potential to have a big impact on agriculture [181]. As a result, the creation of an altered microbiome with the desired properties is required. Many recent investigations have revealed that certain endophytic strains can alter the structure and species richness of plant tissues [182,183]. Very few studies have investigated the internal microbiome of plants for subsequent generations post introduction of a specific strain(s) [181]. Moreover, little research has been carried out on the importance of manipulated microbiomes from disease-suppressive soils on control of phytopathogens [184]. From a practical standpoint, it would be immensely beneficial to establish microbiomes that are durable and stress-tolerant thereby capable of increasing agricultural output [185]. Finally, plant microbiome bioengineering is an intriguing option for improving a plant’s biological capabilities, an approach that, while still in its infancy, has the potential to be of immense agricultural value [186].

### 5.6. Mycoviruses as Biocontrol Agents

Recently, mycoviruses having capability to infect fungal pathogens are known to have the potential to be used as biological control agents against plant diseases. The mycoviruses were recognized to induce hypovirulence (reduced virulence) in their hosts and this notion elicited great interest in characterization of viruses from phytopathogenic fungi as being utilized as biocontrol agents [94,187,188]. Following this information, scores of mycoviruses started to be worked upon and Garcia-Pedrajas et al. [187] further reports that majority of viruses from filamentous fungi possess either double-stranded RNA (dsRNA) genomes or positive sense (+) single-stranded RNA (ssRNA) genomes with dsRNA replicative intermediates, and can possess a capsid forming true virions or be sometimes capsidless. Surprisingly, numerous plant pathogenic fungi are found to harbor mycoviruses, that reduce the virulence of their fungal host [189]. Lacking extracellular transmission route to other isolate, mycoviruses are transmitted primarily through hyphal anastomosis or via conidia in vertical transmission while giving rise to progenies also [187]. Transmission efficiencies are dependent on both the fungal host and the infecting virus and hence it was possible to utilize artificial transfection methods to infect a variety of fungi, thus expanding their possible use to the control of pathogens other than those where they were identified [187]. Although hypovirulence-associated mycoviruses are those mycoviruses that reduce the pathogenicity of their inhabiting fungal hosts. However, it is difficult to transmit these mycoviruses easily between vegetatively incompatible groups and hence it has been difficult to develop commercial mycovirus biocontrol strategies for these phytopathogenic fungi [188].

## 6. Conclusions

Finally, the ever-growing demand for food has led to dependence on chemicals in agriculture that are hazardous to human health. These chemical-based formulations not only create ecological imbalance but also result in ecotoxicity. Organic methods of farming are preferred for sustainable agriculture, but their use incurs high costs that make them inaccessible for most farmers in poor countries. The adoption of diverse biocontrol methodologies, such as those used in organic agriculture, to control plant diseases is environmentally benign, relatively inexpensive, harmless, and has enough potential to significantly boost plant production. As a result, these biocontrol techniques offer enormous benefits for successful rhizosphere management for a sustainable agriculture. The ultimate goal for biocontrol agents is to integrate microbial biofertilizers, biocontrol microorganisms, phages, and phage-based technologies and cocktails, biocontrol yeasts, algae, and cyanobacteria, optimized microbiomes, genetically modified biocontrol techniques, and microbiome engineering. An intelligent experimental trial using a combinatorial approach utilizing all the resources from the strategies discussed would invariably provide enormous leads that could be harnessed by the field plant growers to combat plant diseases. At present, this is an under-researched subject that has the potential to increase crop yields while also addressing food security in an environmentally safe and sustainable manner.

## Figures and Tables

**Table 1:** Examples of plant pathogens and their biocontrol strategies.

Pathogen	Host	Biocontrol Strategies	References
*Phytophthora sojae*, *Pythium heterothallic*, *Pythium irregulare*, *Pythium sylvaticum*, and *Pythium ultimum*	*Glycine max*	*Pseudomonas* water derived strain, 06C 126, effectively inhibited oomycetes	[22]
Soilborne fungal pathogens	Pulses, grapes, cotton, onion, carrot, peas, plums, maize, apple, etc.	The fungal genus *Trichoderma* has biocontrol activity against fungi and nematodes	[23]
*Salmonella* sp., *Staphylococcus aureus*, *Escherichia coli*, *Mycobacterium tuberculosis*, *Shigella* sp., *Listeria monocytogenes* and *Pseudomonas aeruginosa* along with bacteria like *Yersinia pestis, Burkholderia mallei*, *Francisella tularensis*, *Brucella* sp. and *Bacillus anthracis* that pose a bioterrorism risk		Bacteriophage and natural extracts	[24]
Phytopathogenic microorganisms in agriculture or even in other areas		Endophytic *Bacillus toyonensis* BAC3151	[25]
Phytopathogenic fungi		*Trichoderma* spp. potential biocontrol agents	[26]
*Phytophthora* spp. and *Pythium* spp.	Aquaponics	Antagonistic microorganisms	[27]
Soil-borne pathogens		Pathogen-suppressing microorganisms	[28]
Broad range of plant pathogens		Antibiotics, lipopeptides, and enzymes with antagonistic properties against a range of plant pathogens are produced by *Bacillus* species. These bacteria also influence resistance development in plants and stimulate plant growth	[29]
*Ralstonia solanacearum*, *R. pseudo solanacearum*, and *R. syzygii* subsp. *indonesiensis* causative agents of bacterial wilt	Hosts include tomato, potato, banana, tobacco, and peanuts. Losses range from 100% in banana, 90% in potato and tomato and around 20–30% in peanuts and tobacco	Bacteriophage-based bacterial wilt biocontrol methods	[30]
Fungal and bacterial phytopathogens	Many crops	*Streptomyces* spp. as Endophytes mediated biocontrol of phytopathogens	[31]
Pathogens in the crop residues	Cereal crops	Microbiome-based biocontrol strategies	[32]
Fungal pathogens	Cereal crops	*Streptomyces* species produce a range of secondary metabolites that can inhibit the growth of phytopathogens	[33]
Plant fungal pathogen		Improved control obtained with by combinations of fungicides and BCAs (*Trichoderma* spp. or *Bacillus* spp.,)	[34]
Diseases caused by fungi, bacteria, viruses, viroids, nematodes, and oomycetes	*Citrus* sp.	Employment of antagonists produced by *Bacillus* sp. offers superior capacity to restrict diseases in citrus plants	[35]
*Rhizoctonia solani* that induces stem canker, *Fusarium solani* causes tubers dry rot, and black scurf and *Alternaria solani* that induces early blight	Potato	Endophytic bacteria from Romanian potato tubers isolate 6T4 identified as *B. atrophaeus/subtilis* revealed promising perspectives for biocontrol strategies	[36]
*Fusarium oxysporum* and other phytopathogens	Wheat	*Bacillus amyloliquefaciens subsp. plantarum* XH-9 is a rhizobacterium with antagonistic potential against a variety of phytopathogens. It discharges antibiotics and enzymes that are capable of bringing about hydrolysis in the pathogen	[37]
*Verticillium dahliae* soil borne pathogen	Cotton	Endophytic Fungus *Fusarium solani CEF559* against *Verticillium dahliae* in Cotton Plant	[38]
Fungal Pathogens		*Trichoderma* is a fungal genera having antagonistic activity against disease causing fungal pathogens	[39]
*Fusarium* head blight (FHB)	Wheat	Endophytic *Anthracocystis floculossa P1P1*, *Penicillium olsonii ML37*, *Sarocladium strictum C113L*, and *A. floculossa F63P* exhibit the ability to act as biocontrol agents against FHB	[40]
Fungi *Ustilaginoidea virens*, *Alternaria alternata*, *Fusarium oxysporum*, *Botrytis cinerea*, *Fulvia fulva*, and *Fusarium graminearum*	Tomato	Antifungal metabolites of *Bacillus velezensis* NKG-2	[41]
Bacterial phytopathogen *Pseudomonas syringae* pv. Tomato	Tomato	*Pseudomonas segetis* strain P6 isolated from the rhizosphere has the ability to induce plant growth and inhibit quorum sensing abilities of bacterial pathogens	[42]
Pepper gray mold caused by *Botrytis cinerea*	Pepper	Can be controlled efficiently by the biocontrol mediator *Bacillus velezensis*	[43]
Seed and soil borne pathogens		*Chaetomium globosum* functions as an effective potential biocontrol agent	[44]
Fungal Pathogen		Endophyte and epiphyte microbiome of Grapevine leaf as biocontrol agents against phytopathogen	[45]
Fungal pathogen	*Vitis vinifera*	*Bacillus licheniformis* GL174 culturable endophytic strain isolated from *Vitis vinifera* cultivar Glera	[46]
Species of soil-borne fungal plant pathogens, such as *Cladosporium variabile*, *Rhizoctonia fragariae*, *Phomopsis longicolla*, *Colletotrichum acutatum*, *Aspergillus niger*, *Sclerotinia sclerotiorum*, *Penicillium digitatum*, *Macrophomina phaseolina*, *Trichoderma viride* and *Botrytis squamosa*		Natural wine yeast strains of Saccharomyces and Zygosaccharomyces	[47]
Endophytic fungal parasite of *Moniliophthora perniciosa* causing Witches’ Broom Disease	*Cacao*	Yeasts, such as *Saccharomyces cerevisiae* and *Wickerhamomyces anomalus*	[48]
*Cryphonectria parasitica* causing chestnut blight epidemic	Chestnut	*Mycoviruses*	[49]
Closteroviridae family of plant viruses causing leafroll disease	*Grapevine*	Case based management, such as use of certified planting material, open field foundation block vineyards on virgin soil etc.	[50]
Cucurbit yellow stunting disorder virus, Cucurbit chlorotic yellows virus and Beet pseudo-yellows virus	Vegetable crops	Integrated disease management strategies and using resistant varieties	[51]
*Pythium ultimum*	Chilly, Tomato, Redgram, Chickpea, Soybean, etc.	*Trichoderma viride*, *T. harzianum*, *T. virens* and *Laetisaria arvalis*	[52]
Wilt diseases		*Trichoderma* spp.	[53]

## Data Availability

Not applicable.
